# Managing chondrosarcoma of the epiglottis: a case report

**DOI:** 10.1308/003588412X13373405387258

**Published:** 2012-05

**Authors:** E Maughan, M Pankhania, K Shah, P Gurr

**Affiliations:** ^1^Heatherwood and Wexham Park Hospitals NHS Foundation trust,UK; ^2^Oxford University Hospitals NHS Trust,UK; ^3^Milton Keynes Hospital NHS Foundation Trust,UK

**Keywords:** Chondrosarcoma, Laryngeal Cancer, Epiglottis

## Abstract

Laryngeal chondrosarcomas are a very rare malignancy with less than 150 cases reported in the literature. Of these, the epiglottis is the most unusual primary neoplastic subsite. Uncertainties arise owing to the extremely rare nature of the condition with regard to treatment and investigation for metastases in overtly low grade cases. We present the case of a 62-year-old woman with a low grade chondrosarcoma, arising from the tip of the epiglottis, presenting with dysphagia but no other symptoms.

Only 150 cases of laryngeal chondrosarcoma exist in the world literature. The epiglottis is the least common primary site of tumour with four cases reported worldwide. Over 95% of all chondrosarcomas are low grade and are treated by surgical excision, including conservative surgical laryngectomy options.^1–4^

Diagnostic uncertainties arise due to the extremely rare nature of laryngeal chondrosarcomas. Treatment dilemmas exist regarding the metastatic potential of epiglottic chondrosarcomas compared with chondrosarcomas elsewhere in the body.^2,4,5^

## Case history

A 62-year-old woman presented to the otolaryngology department with a 6-month history of worsening dysphagia, having been referred by her general practitioner under the ‘2-week wait’ initiative for suspected head and neck malignancy. This affected solids but liquids were unaffected. She had no odynophagia or reflux symptoms and her voice was unchanged. There were no lateralising symptoms. She was well systemically and had no significant medical or family history. She had never smoked and drank alcohol only occasionally.

Mouth and oropharyngeal examinations were normal. There were no palpable neck nodes. A flexible nasendoscopy was performed in clinic. This yielded views of a large smooth swelling on the tip of the epiglottis.

### Investigations and treatment

**Figure 1:**
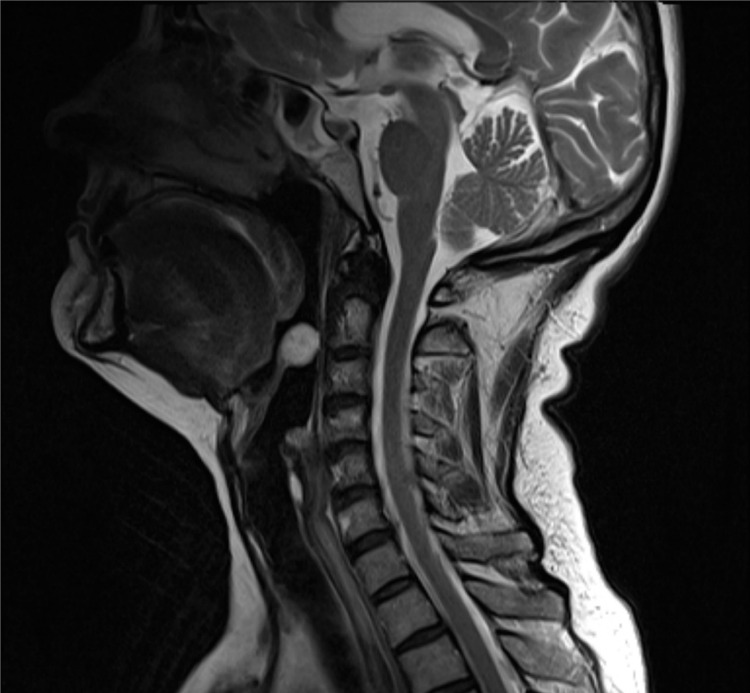
T2 weighted sagittal magnetic resonance imaging showing a smooth, well defined mass arising from the tip of the epiglottis

Magnetic resonance imaging of the neck, performed to delineate the lesion, showed a large, spherical, well-circumscribed mass extending from the tip of the epiglottis, with high signal intensity in T2 weighted images. The lesion represented a mucous retention cyst radiologically (Fig 1). There appeared to be a wide margin of normal epiglottic cartilage separating the mass from the rest of the laryngeal cartilage skeleton.

**Figure 2:**
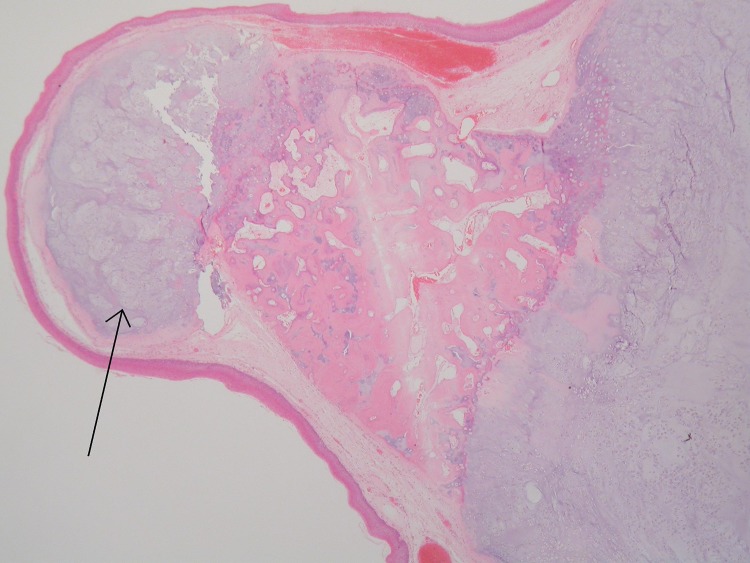
Scanning power photomicrograph showing a focally ossifying cartilaginous tumour underneath the epiglottic squamous epithelium

**Figure 3:**
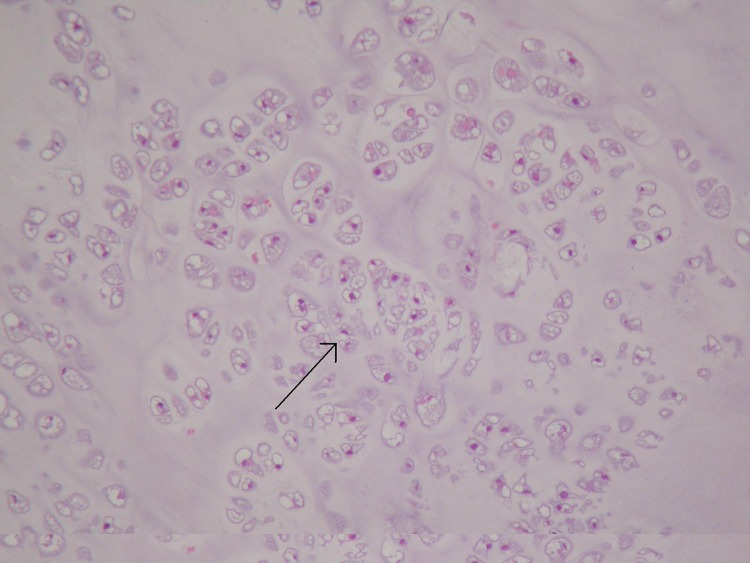
Medium power photomicrograph showing a lobular and patchily hypercellular proliferation of atypical chondrocytes

**Figure 4:**
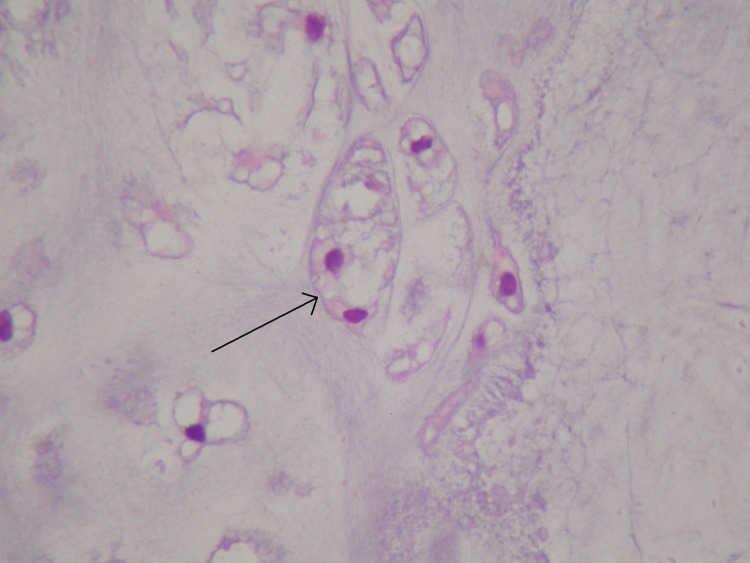
High power photomicrograph showing atypical binucleate chondrocytes in a single lacuna

Laser excision biopsy of the lesion was carried out uneventfully. The swelling was felt to resemble a mucous retention cyst, measured 11mm × 11mm × 6mm and was sent for histological analysis. Histologically, the cyst was smooth surfaced and firm macroscopically. Microscopy demonstrated a polypoid, neoplastic mass covered by non-dysplastic squamous epithelium. The core contained a lobulated proliferation of cartilaginous tissue with focal ossification and central focal degenerative change. There was little nuclear polymorphism seen (Figs 2–4).

There was diagnostic uncertainty as to whether the mass represented a benign or malignant lesion. Consequently, a tissue block and haematoxylin and eosin stain were sent for a second opinion. The provisional diagnosis of chondrosarcoma was supported: ‘Although a benign chondroma has been considered, given the areas of increased cellularity and focal binucleated chondrocytes, this is best regarded as a low grade chondrosarcoma.’

Although the tumour closely approached the base of the specimen, it was separated from the margin by a thin rim of connective tissue and appeared to have been excised completely. Although the neoplasm appeared low grade, further partial excision of the epiglottis was performed to confirm a clear margin of excision.

### Outcome and follow-up

Excision margins were confirmed as clear. There has been no recurrence at 18 months post-excision and follow-up is planned for 5 years. There was clinical uncertainty as to the appropriate follow-up duration due to the lack of evidence base. Following the second excision of tissue from the epiglottis to confirm clearance, the patient reported occasional symptoms of aspiration but these resolved after six months, with speech and language therapy advice and intervention.

## Discussion

Laryngeal chondrosarcomas are an extremely rare form of tumour that show a wide variability in behaviour, with high grade tumours acting in a locally aggressive fashion as well as spreading via haematogenous and lymphatic routes to the lungs, kidney, spleen and cervical vertebrae. Low grade chondrosarcomas behave more like benign chondromas.^5^

The epiglottis is the rarest subsite of this tumour, with four cases reported in the literature. Of these, only one case went on to develop metastasis and was confirmed to be high grade at histological analysis.^2^

Five-year survival and metastatic behaviour varies with grade of neoplasm. Low grade chondrosarcoma has a 90% five-year survival and 0% rate of metastasis. High grade chondrosarcoma has a much lower five-year survival rate of 43% and a 71% metastasis rate.^5^

There has been some controversy in the past regarding the need to completely excise low grade chondrosarcomas in the head and neck, given that there is a negligible risk of metastasis. More recent opinion advocates complete excision, supplemented with radiotherapy in cases where excision might be difficult.^5^

## Conclusions

The rarity of such epiglottic lesions reinforces the need for histological investigation of excised specimens. A second opinion should be considered readily in cases where malignancy needs to be established. Histology will form the basis for ongoing management and follow-up. Discussion in a multidisciplinary setting is therefore advocated to ensure accurate diagnosis, appropriate management and intervention where necessary.
